# Association between cognitive impairment patient with solid cancer and insulin resistance

**DOI:** 10.1186/s13104-019-4739-5

**Published:** 2019-10-28

**Authors:** Kenji Gonda, Kenji Yaginuma, Yuichi Rokkaku, Shoichiro Horita, Yuko Maejima, Kenju Shimomura

**Affiliations:** 1 Department of Bioregulation and Pharmacological Medicine, Fukushima Medical University School of Medicine, 1 Hikarigaoka, Fukushima, 960-1295 Japan; 2 Daido Obesity and Metabolism Research Center, 123 Daido, Naha, Okinawa 902-0066 Japan; 3 Center for Medical Genetics and Immunology, Fukushima Medical University School of Medicine, 1 Hikarigaoka, Fukushima, 960-1295 Japan; 4 Japan Community Healthcare Organization Nihonmatsu Hospital, 1-553 Narita, Nihonmatsu, Fukushima 964-8501 Japan

**Keywords:** Cognitive impairment, Solid cancer, HOMA-B, HOMA-IR

## Abstract

**Objectives:**

In an aging population, an increase in the number of elderly cancer patients with cognitive impairment is expected. The possible association between cancer and cognitive impairment is important to elucidate, because it can have a serious impact on quality of life. Here, we focused on glucose metabolism as a factor that links cancer and cognitive impairment.

**Results:**

Thirteen subjects with solid cancers and cognitive impairment were recruited. As a control group, 14 subjects with cognitive impairment alone and 8 subjects with cancer alone were recruited. A Homeostatic Model Assessment of Insulin Resistance (HOMA-IR) and that of β-cell function (HOMA-B) were used. In comparison with patients with solid cancer alone, those with cognitive impairment alone and those with both cancer and cognitive impairment had increased HOMA-IR values. Insulin resistance was increased in patients with cognitive impairment alone and those with both cognitive impairment and solid cancer than in patients without cognitive impairment; however, β-cell function was not affected. The present data indicated that elderly cancer patients with high HOMA-IR score may be at a relatively high risk for developing cognitive impairment. Furthermore, early treatment to reduce insulin sensitivity may prevent cognitive impairment.

## Introduction

An increase in the number of elderly patients with cancer and/or cognitive impairment is inevitable in an aging population. Cognitive impairment has recently been shown to affect up to 30% of patients with cancer [1–5] and can have a serious impact on the quality of life of both patients and families. However, the association between cognitive impairment and cancer remains unknown, and there has been no effective treatment for such patients. Recent studies have indicated that diabetes contributed to the development of cognitive impairment, such as Alzheimer’s disease [6]. A number of reports have also indicated that hyperglycemia is a contributing factor to the progression of cancer [7]. Therefore, hyperglycemia or glucose intolerance may be the key factor that links the development of cognitive impairment in patients with cancer [8]. Hyperglycemia can be induced by two different mechanisms; one is reduction of insulin secretion from pancreatic β-cells, and the other is increased insulin resistance in the target organs. The well-known cause of diabetes in a majority of cases in Asia is reduced insulin secretion; whereas that for the United States and Europe is insulin resistance. However, little is known on the contribution of hyperglycemia to cognitive impairment and cancer. Because the number of elderly cancer patients with cognitive impairment is expected to increase, understanding the underlying mechanism that links both diseases is important. In this study, we focused on the aspect that may link hyperglycemia with cognitive impairment and cancer. We applied a homeostasis model assessment (HOMA) to assess insulin resistance (HOMA-IR) and β-cell function (HOMA-B) in elderly patients with solid cancer (i.e., esophagus, gastric, colon, bile duct, prostate, breast, lung and ovary) and those with cognitive impairment, as well as in patients with both cancer and cognitive impairment.

## Main text

### Methods

#### Patient information

A total of 13 subjects (7 men and 6 women) with an average age of 85 years and who had solid cancers and cognitive impairment were recruited (Table 1). For the control group, we recruited 14 subjects (6 men and 8 women) with an average age of 86 years and who had cognitive impairment alone and 8 subjects (5 men and 3 women) with an average age of 88 years and who had cancer alone. For the 8 patients with cancer alone, malignancy was based on tissue diagnosis.

**Table 1:** The clinical and laboratory features of patients with both cognitive impairment and cancer

Age	Gender	Cognitive impairment	Cancer	BS	IRI	IR	%B
80s–90s	M	Alzheimer	Esophagus	204	6.1	0.95	16.5
80s–90s	M	Alzheimer	Stomach	192	11.6	1.77	30
80s–90s	F	Cerebrovascular	Stomach	213	12.2	1.91	26
70s–80s	F	Cerebrovascular	Colon	190	11.9	1.81	31.1
90s–100s	M	Cerebrovascular	Colon	273	10.4	1.91	15.1
80s–90s	M	Alzheimer	Colon	195	8.5	1.31	23
80s–90s	F	Alzheimer	Pancreas	318	10.3	2.38	11.8
80s–90s	M	Alzheimer	Bile duct	191	11.2	1.71	29.4
90s–100s	M	Cerebrovascular	Prostate	220	13.3	2.1	26.3
70s–80s	F	Alzheimer	Breast	110	12.7	1.72	90.4
90s–100s	M	Alzheimer	Lung	198	16.3	2.48	37
80s–90s	F	Parkinson	Ovary	200	15.4	2.35	34.8
70s–80s	F	Alzheimer	Vulvar	275	12.2	2.25	17

*BS* blood sugar (mg/dL), *IRI* insulin (μU/mL), *IR* Homeostatic Model Assessment of Insulin Resistance (HOMA-IR), *%B* Homeostatic Model Assessment of β-cell Function (HOMA-B) (%)

#### Research methods

The Mini-Mental State Examination (MMSE) [9–11] and the Revised Hasegawa’s Dementia Scale (HDS-R) tests were used for cognitive assessment of the patients [12].

Blood samples were collected at 07:00 a.m. after overnight fasting to measure fasting plasma glucose and fasting insulin levels.

#### Statistical analysis

The HOMA-IR and HOMA-B values were calculated using a HOMA calculator, which was available on the Diabetes Trials Unit website (http://www.dtu.ox.ac.uk). All the values were expressed as average + SD.

### Results

#### Comparison of β-cell function

**Fig. 1:**
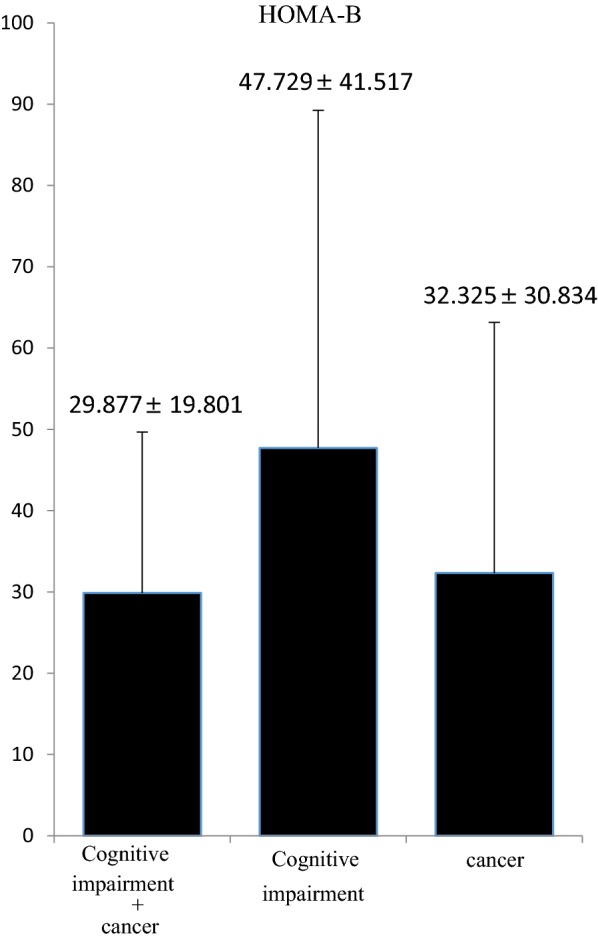
Results of HOMA-B evaluation. The HOMA-B values of patients with cancer alone, cognitive impairment alone, and both are not different. Data are presented as average ± SD

The HOMA-B values were not different among patients with cognitive impairment alone (47.729% ± 41.517%), cancer alone (32.325% ± 30.834%), and both (29.877% ± 19.801%) (Fig. 1).

#### Comparison of insulin resistance

**Fig. 2:**
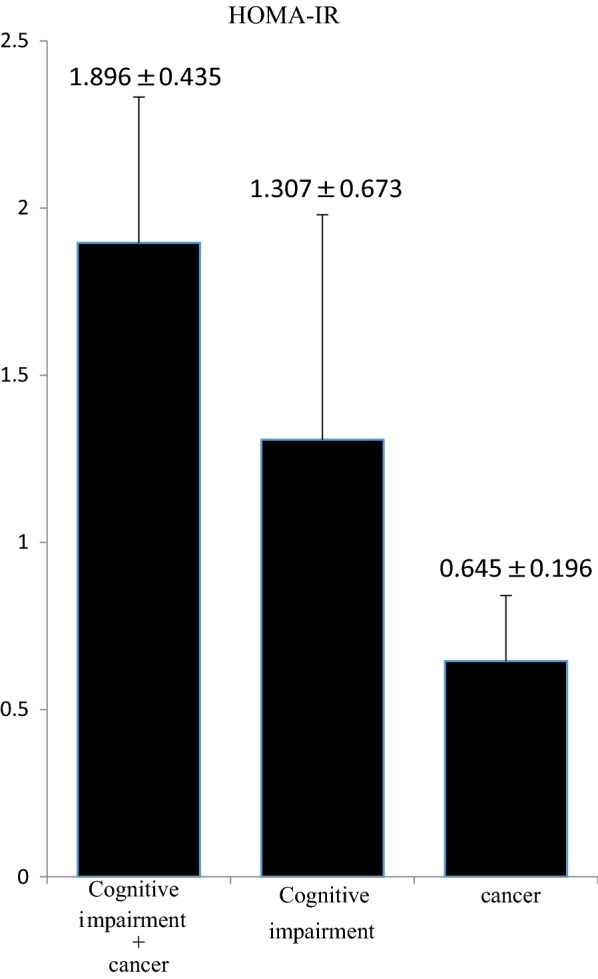
Results of HOMA-IR evaluation. The HOMA-IR values are higher in patients with cognitive impairment alone and in those with both cognitive impairment and cancer than in patients with cancer alone. Data are presented as average ± SD

Insulin resistance was different among the groups. In particular, the HOMA-IR values were higher in patients with cognitive impairment alone (1.307 ± 0.673) and in those with both cognitive impairment and cancer (1.896 ± 0.435) than in those with cancer alone (0.645 ± 0.196) (Fig. 2).

#### Clinical features of the patients and the relationship between blood sugar (BS) and immune-reactive insulin (IRI) level

Compared with patients with cognitive impairment alone, those with both cognitive impairment and cancer had higher levels of BS (213.769 ± 51.134 vs. 160.429 ± 51.831 mg/dL) and IRI (11.700 ± 2.653 vs. 8.993 ± 4.739 μU/mL) (Table 1).

### Discussion

In the present study, we have shown that insulin resistance, based on the HOMA-IR, was increased in patients with cognitive impairment, regardless of the presence of solid cancer, compared with that in cancer patients without cognitive impairment. The similar HOMA-IR values between patients with cognitive impairment alone and those with both cognitive impairment and cancer suggested that the presence of solid cancer itself did not contribute to the development of insulin resistance in cancer patients.

To date, many studies have indicated the relationship between insulin resistance and cancer development or progression [13]. However, in the present study, the HOMA-IR was significantly lower in patients with cancer alone than in those with cognitive impairment alone. Therefore, the contribution of insulin resistance to cancer development and progression was not evident, and further studies are required to validate these findings. On the other hand, recent epidemiologic and basic scientific investigations have suggested an association and common pathologic mechanisms between hyperglycemia and cognitive impairment, including Alzheimer’s disease [7]. Interference in the insulin signal processing in the brain has been indicated as the mechanism for the development of cognitive impairment in diabetic patients. Wan et al. reported that insulin induced functional postsynaptic gamma-aminobutyric acid (GABA) receptors in the brain [14]. Furthermore, low insulin sensitivity was reported to contribute to decreased acetylcholine synthesis, which leads to Alzheimer’s disease [15]. Our present data suggested that cancer patients are not exempted from developing hyperglycemia due to low insulin sensitivity, which induces cognitive impairment. However, based on the similar HOMA-IR values between patients with cognitive impairment alone and those with both cancer and cognitive impairment, insulin resistance may not be the sole contributing factor to the development of cognitive impairment in patients with solid cancers. Interestingly, a majority of Japanese diabetic patients have been known to have insulin secretion deficiency but not insulin resistance [16]. Our present data implied the importance of HOMA-IR measurement in elderly cancer patients, because those with high HOMA-IR scores may be at a high risk for developing cognitive impairment and may benefit from early treatment, such as the use of biguanide, to reduce insulin sensitivity. However, further studies are required to investigate the effects of biguanide on the development of cognitive impairment in elderly cancer patients.

In summary, our results suggested that insulin resistance but not β-cell function was increased in patients with cognitive impairment alone and those with both cognitive impairment and solid cancer than in cancer patients without cognitive impairment. Elderly cancer patients with insulin resistance may be at a high risk for developing cognitive impairment, which may be prevented by early treatment that reduces insulin sensitivity.

## Limitations

Due to the small number of subjects, this study cannot show a cause–effect relationship strictly. Social acceptance and recall bias were also possible confounding factors.

## Data Availability

The dataset in the current study is available from the corresponding author upon request.

## Abbreviations

HOMA-IR: homeostasis model assessment was used to assess insulin resistance
HOMA-B: homeostasis model assessment was used to assess β-cell function
BS: blood sugar
IRI: immune-reactive insulin
